# Phenotypic characterization of mucosal-associated invariant T cells in visceral leishmaniasis and HIV co-infection

**DOI:** 10.3389/fimmu.2026.1882545

**Published:** 2026-08-11

**Authors:** Victoria Hellena Silva Pereira, Gustavo Augusto Fonseca Nogueira, Luana Borges-Fernandes, Silvia Hees de Carvalho, Adelina Machado de Carvalho Nogueira, Argus Leão Araújo, Joaquim Pedro Brito-de-Sousa, Juliana Assunção Rodrigues, João Paulo Resende Magalhães Toledo, Vanessa Caroline Randi Magalhães, Jairo Campos de Carvalho, Livia Fulgencio da Cunha, Jordana Grazziela Alves Coelho-dos-Reis, Andréa Teixeira-Carvalho, Olindo Assis Martins-Filho, Gabriel Macedo Costa Guimarães, Vanessa Peruhype-Magalhães

**Affiliations:** 1 Grupo Integrado de Pesquisa em Biomarcadores, Instituto René Rachou, Fundação Oswaldo Cruz (Fiocruz Minas), Belo Horizonte, Brazil; 2 Hospital Eduardo de Menezes, Fundação Hospitalar do Estado de Minas Gerais (FHEMIG), Belo Horizonte, Brazil; 3 Department of Microbiology, Universidade Federal de Minas Gerais (UFMG), Belo Horizonte, Brazil; 4 ProsPERA-UFMG Research Group (Prospecção e Pesquisa em Resposta Imune e Antivirais), Universidade Federal de Minas Gerais (UFMG), Belo Horizonte, Brazil

**Keywords:** flow cytometry, HIV co-infection, immune exhaustion, MAIT cells, visceral leishmaniasis

## Abstract

**Introduction:**

Mucosal-associated invariant T (MAIT) cells are unconventional T lymphocytes with innate-like antimicrobial responsiveness. Despite their recognized role in bacterial and viral infections, their involvement in protozoal diseases, particularly visceral leishmaniasis (VL) and HIV co-infection, remains poorly understood.

**Methods:**

A cross-sectional study evaluated the immunophenotypic profile of circulating MAIT cells in visceral leishmaniasis patients (VL, n = 8), VL–HIV co-infection patients (VL–HIV, n = 10), VL–HIV coinfected patients under liposomal amphotericin B prophylactic treatment (VL–HIV_(T)_, n = 9), and reference groups comprising HIV-mono-infected (HIV, n = 11) and non-infected (NI, n = 11) individuals. Multiparameter flow cytometry assessed MAIT cell frequency, subset distribution (CD4^+^, CD8^+^ and CD4^-^CD8^-^), and the expression of activation, costimulatory, cytotoxic, exhaustion and memory markers. Integrative correlation network analysis was performed to evaluate immunological interactions among MAIT cell markers.

**Results:**

MAIT cell frequencies were significantly reduced in VL and VL–HIV, predominantly affecting the CD8^+^ subset. Fold-change analyses demonstrated increased activation and cytotoxicity signatures in VL and VL–HIV patients, with marked upregulation of CD69, CD38, NKG2D, and CD107A, concomitant with increased expression of inhibitory receptors (PD-1, TIM-3, LAG-3), particularly in CD8^+^ and CD4^−^CD8^−^ MAIT cells. In contrast, VL–HIV_(T)_ patients exhibited a decrease in this hyperactivated/exhausted phenotype and partial restoration of central memory-associated features, suggesting immune rebalancing under treatment. Integrative correlation network analysis revealed marked differences of MAIT cell immunological interactions. VL and VL–HIV groups displayed reduced network density, decreased selective axis connectivity, and fragmentation of interactions among activation, cytotoxicity, and memory nodes, consistent with immune disorganization. Conversely, treated VL–HIV_(T)_ patients exhibited increased connectivity and re-establishment of coordinated interactions between functional markers, indicating partial re-establishment of the MAIT cell immunological network architecture.

**Discussion:**

Together, VL and VL–HIV co-infection are associated with quantitative depletion and qualitative dysregulation of circulating MAIT cells, characterized by concurrent hyperactivation and exhaustion, coupled with immunological network fragmentation, while liposomal amphotericin B treatment appears to promote partial immunological reorganization.

## 1 Introduction

Visceral leishmaniasis (VL), caused by parasites of the *Leishmania donovani* complex, predominantly *Leishmania infantum* in Brazil (1–6), is the most severe manifestation of leishmaniasis and remains a major global health challenge due to its high morbidity and mortality (3, 5, 7–10). Paradoxically, despite recent stabilization in incidence (5, 6, 11), VL-associated lethality has increased over time, driven by delayed diagnosis, therapeutic constraints, the geographic expansion of transmission vectors (12, 5, 6, 13), and the emergence of HIV co-infection that profoundly reshaped the epidemiology and clinical course of VL, with coinfected patients showing substantially higher lethality and relapse rates than those with VL alone (14, 15);. In Brazil, nearly 19% of VL cases occur in people living with HIV, in whom mortality is markedly elevated (5, 6, 15–17). This excess risk is underpinned by bidirectional pathogenic interactions: HIV-mediated immunosuppression increases susceptibility to *Leishmania* infection and accelerates disease progression, while chronic immune activation induced by the parasite enhances HIV replication (15, 16, 18–20). Together, these processes establish a self-reinforcing cycle that exacerbates disease severity and complicates clinical management, rendering VL–HIV co-infection one of the most challenging scenarios in tropical medicine (16, 18, 19).

The VL–HIV co-infection is characterized by prominent immunological complexity (16). Macrophage dysfunction, T lymphocytes activation, exhaustion and senescence, and massive CD4^+^ T-cell depletion are observed in coinfected patients (21–23). Furthermore, in VL–HIV co-infection, the “cytokine storm”, severe systemic inflammatory syndrome, characterized by secretion of IL-10, IL-16 and TNF, is more pronounced (24, 25).

Non-conventional T cells (innate-like T lymphocytes) also play an important role in the immunopathology of VL. For the past 10 years, our group has been studying the role of Mucosal-associated invariant T (MAIT) cells in VL (26, 27). These cells recognize riboflavin-derived microbial metabolites and are highly enriched in peripheral blood and barrier tissues, particularly the liver, positioning them as key sentinels in antimicrobial immunity (28, 29). MAIT cells are a conserved population of innate-like T lymphocytes characterized by a semi-invariant αβ T cell receptor and restriction by the monomorphic MR1 molecule (26, 27).

Both VL and HIV independently disrupt MAIT cell homeostasis. VL is associated with reduced MAIT cell frequencies, heightened activation, and impaired effector function, correlating with clinical indicators of disease severity. VL is linked to reduced circulating MAIT cells with heightened activation and MR1-dependent effector responses that track markers of liver injury, anemia and organ involvement (30, 31). HIV causes early, persistent MAIT depletion with activation and functional exhaustion, only partly corrected by antiretroviral therapy (32–41). In a similar vein, these alterations are hypothesized to result from a combination of chronic immune activation, activation-induced cell death, microbial translocation, and altered tissue homing/redistribution of MAIT cells, as suggested by studies of MAIT cell activation, localization, and dysfunction in HIV and other chronic infections (32, 33, 39, 42).

Despite these advances, the impact of VL–HIV co-infection on MAIT cell biology remains poorly characterized (22, 43, 44). Understanding the phenotypic and functional landscape of MAIT cell in this context is critical for elucidating the immunopathogenesis of co-infection and for identifying potential immunological targets for therapeutic intervention (30, 31, 45, 46). In this study, we conducted an in-depth phenotypic characterization of MAIT cells across individuals with VL, VL–HIV co-infection and VL–HIV patients in liposomal amphotericin B prophylaxis, evaluating markers of activation, co-stimulation, cytotoxicity, exhaustion, and immunological memory. Our central hypothesis is that VL–HIV co-infection imposes a compounded and distinct pattern of MAIT cell dysregulation, and that liposomal amphotericin B prophylactic treatment supports phenotypic and functional reestablishment of MAIT cells.

## 2 Population, materials and methods

### 2.1 Study population

This observational descriptive investigation was conducted at René Rachou Institute, Fiocruz-Minas and Eduardo de Menezes Hospital (HEM), a state reference center for infectious diseases in Belo Horizonte, Brazil. Eligible participants were adults (18–65 years) of both sexes who provided written informed consent. Participants were stratified into five groups: visceral leishmaniasis (VL, n = 8), VL–HIV co-infection (VL–HIV n = 10) and VL–HIV coinfected patients under liposomal amphotericin B prophylactic treatment (VL–HIV_(T)_, n = 9), as well as reference groups comprising non-infected individuals (NI, n = 11) and HIV-mono-infected individuals (HIV, n = 11). HIV-mono-infected individuals were included only if they was under regular antiretroviral therapy (ART; Dolutegravir DTG + Tenofovir TDF + Lamivudine 3TC) and presented controlled infection, defined by undetectable viral load and stable clinical condition at the time of sample collection, with a median CD4^+^ T-cell count of 331 (22|1306) cells/mm³. VL-HIV patients were under regular ART administered at standard doses, with a median of viral load of 24.541 (68|1.680.000) copies/mL and median CD4^+^ T-cell count of 165 (11|701) cells/mm³. VL-HIV_(T)_ patients under regular antiretroviral therapy and receiving regular secondary prophylaxis with liposomal amphotericin B, consisting of intravenous administration of 3 mg/kg every two weeks, according to clinical protocols for immunocompromised individuals. These patients exhibited predominantly undetectable viral load and median CD4^+^ T-cell count of 164 (70|336) cells/mm³. VL diagnosis was confirmed based on clinical assessment and immunochromatographic rapid test, Leishmaniose VHBio (Bioclin, Quibasa, MG, BR) or PCR method according to Brazilian Ministry of Health guidelines (47). HIV infection was confirmed using immunochromatographic rapid tests for HIV-1/2, manufactured by Bio-Manguinhos, Fiocruz, using either finger-prick whole blood or oral fluid (48). This study was approved by the Research Ethics Committees of René Rachou Institute, Fiocruz-Minas (CAAE: 64332422.1.0000.5091) and FHEMIG/HEM (CAAE: 64332422.1.3001.5124). All participants provided written informed consent in accordance with the Declaration of Helsinki and Resolution 466/2012 of the Brazilian National Health Council.

### 2.2 Blood sampling

**Figure 1:**
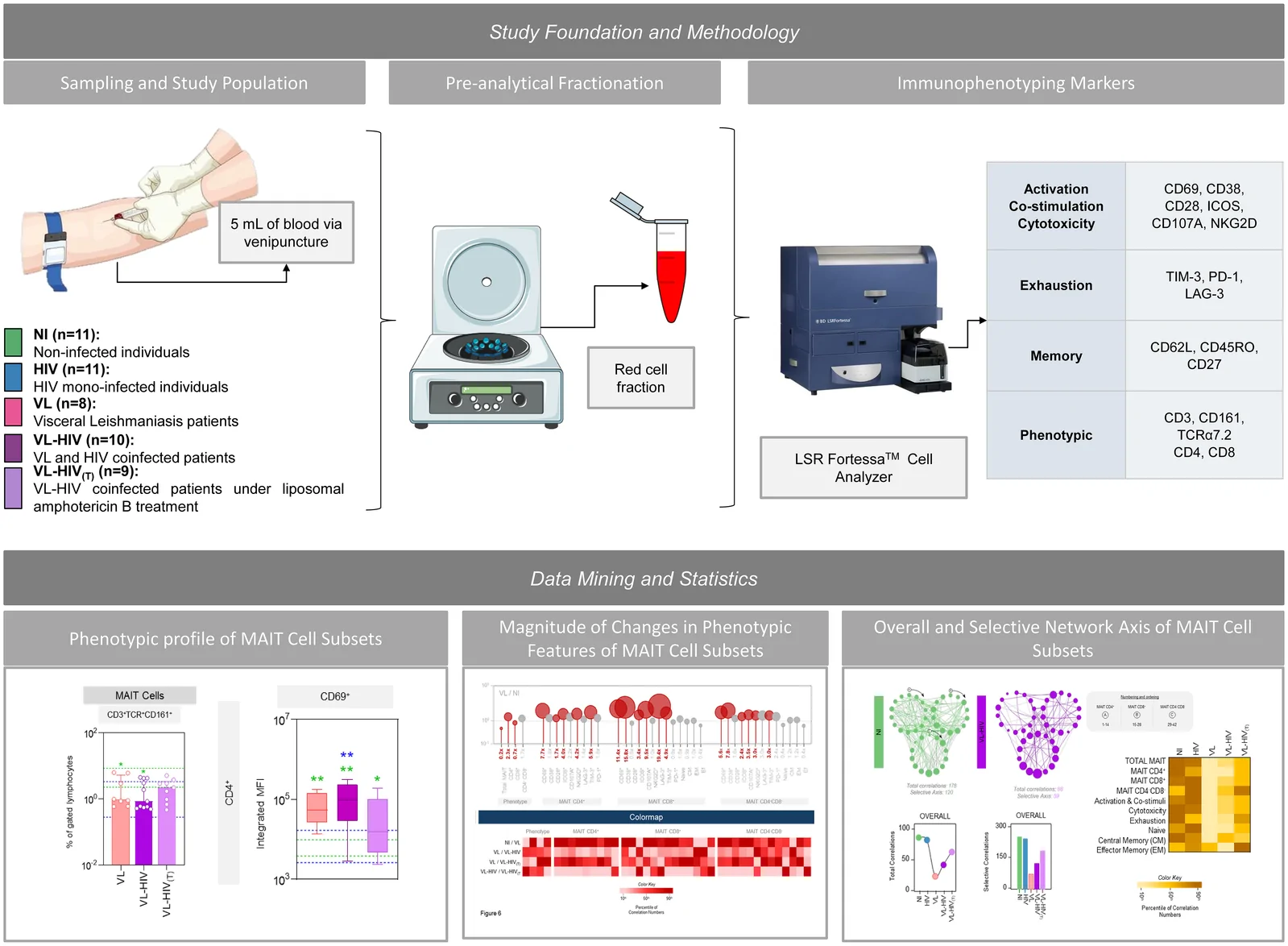
Study foundation and methodology. An overview of sampling, pre-analytical steps, experimental procedures, data mining and statistical analysis employed in the present investigation. The study population comprised individuals with different conditions: VL (
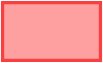
, VL, n = 8), VL–HIV co-infection (
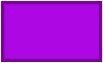
, VL–HIV, n = 10) and VL–HIV coinfected patients under liposomal amphotericin B treatment (
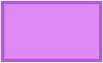
, VL–HIV_(T)_, n = 9), as well as reference groups comprising HIV-mono-infected individuals (HIV, n = 11) and non-infected individuals (NI, n = 11). Whole blood samples (5 mL) were collected from each subject in vacuum system containing sodium citrate as anticoagulant. Plasma and cellular fractions were separated by density gradient centrifugation. The immunophenotypic characterization of MAIT cells was performed by flow cytometry using a panel of markers including activation/co-stimulation and cytotoxicity markers (CD69, CD38, CD28, CD107A, NKG2D); exhaustion markers (TIM-3, PD-1, LAG-3); memory markers (CD62L, CD45RO, CD27); and phenotypic markers defining populations (CD3, CD161, TCRα7.2, CD4 and CD8). Flow cytometric acquisition was performed using an LSR Fortessa Cell Analyzer (BD Biosciences). Data mining and statistical analysis included characterization of overall MAIT cell profiles across study groups, quantification of MAIT cell activation/co-stimulation and cytotoxicity, exhaustion and memory status compartments, assessment of magnitude of phenotypic, activation, cytotoxic, exhaustion and memory alterations in circulating MAIT cell subsets, construction of integrative networks based on total correlations among analyzed parameters, and identification of selective network axes discriminating between study groups. Statistical significance was assessed using the Mann–Whitney U test. * p < 0.05 and ** p < 0.01.

Peripheral blood samples were collected at HEM and immediately processed at the Grupo Integrado de Pesquisas em Biomarcadores, René Rachou Institute, Fiocruz-Minas, Belo Horizonte, Brazil. Blood samples (5 mL) were obtained by venipuncture using vacuum system in tubes containing EDTA. Complete blood counts were performed using the Sysmex XN-550 automated hematology analyzer. All pre-analytical steps were carried out following standardized operating procedures, following good laboratory practices. An overview of sampling, pre-analytical steps, experimental procedures, data mining and statistical analysis employed in the present investigation is presented in **Figure 1**.

### 2.3 Evaluation of the immunophenotypic profile of MAIT cells

For immunophenotyping, 300 μL of peripheral blood were transferred to 5 mL polystyrene tubes containing predefined antibody mix, prepared in phosphate-buffered saline (PBS; 137 mM NaCl, 2.7 mM KCl, 10 mM Na_2_HPO_4_, 1.8 mM KH_2_PO_4_, pH 7.4) added of 5% bovine serum albumin (BSA) for MAIT cell analysis. Surface markers included: CD3 (clone SK7, BV650, BD Biosciences), CD161 (clone DX12, BV605, BD Biosciences), and TCRVα7.2 (clone 3C10, FITC, BD Biosciences) for MAIT cell identification; CD4 (clone RPA-T4, APC-H7, BD Biosciences) and CD8 (clone SK1, AF700, BD Biosciences; clone RPA-T8, BV510, BD Biosciences) for subset characterization; CD69 (clone FN50, BV510, BD Biosciences) and CD38 (clone HIT2, PE, BD Biosciences) for activation profile; CD28 (clone CD28.2, BV711, BD Biosciences), and ICOS (clone DX29, PE, BD Biosciences) for co-stimulation profile; CD107A (clone H4A3, BV421, BD Biosciences) and NKG2D (clone 1D11, BV711, BD Biosciences) for cytotoxicity function; TIM-3 (clone 7D3, BV786, BD Biosciences), PD-1 (clone MIH4, APC, BD Biosciences), and LAG-3 (clone T47-530, BV421, BD Biosciences) for exhaustion profile; and CD62L (clone DREG-56, BV421, BD Biosciences), CD45RO (clone UCHL1, BV786, BD Biosciences), and CD27 (clone M-T271, AF700, BD Biosciences), for memory profiles. All samples were vortexed and incubated for 30 minutes at room temperature in the dark. Subsequently, 3 mL of FACS Lysing Solution (BD Biosciences) were added under gentle vortexing, followed by 10 minutes of incubation. Cells were centrifuged at 380 x g for 7 minutes at 18 °C, washed with 2 mL of PBS, centrifuged again under the same conditions, and resuspended in 250 µL PBS. Samples were kept up to 12 hours at 4 °C until acquisition. Flow cytometric acquisition was performed using an LSR Fortessa™ Cell Analyzer (BD Biosciences), with 200,000 CD3^+^ T lymphocytes acquired per sample within the lymphocyte gate.

### 2.4 Data analysis

**Figure 2:**
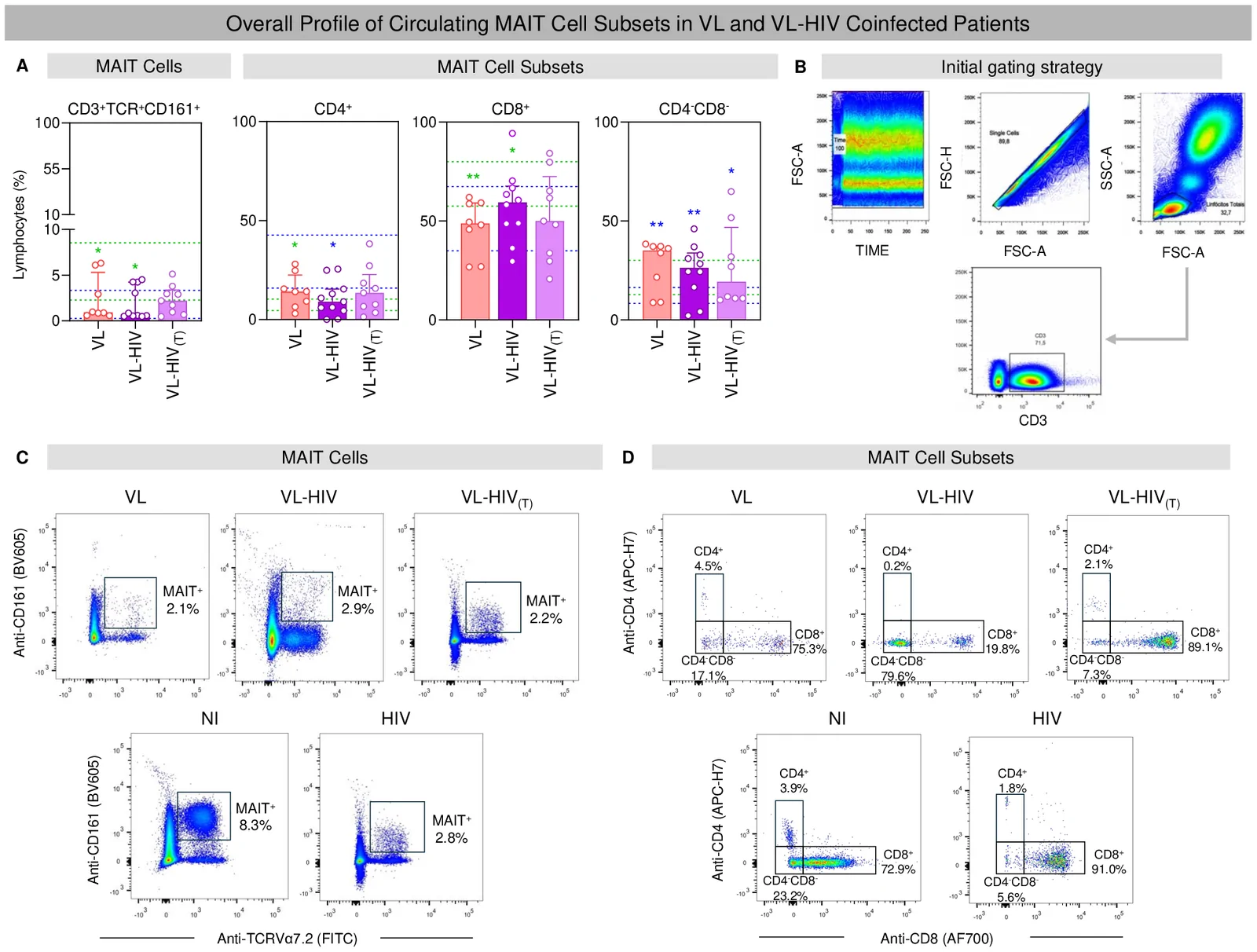
Overall profile of circulating MAIT cell subsets in VL and VL–HIV coinfected patients. **(A)** The overall profile of MAIT cells (CD3^+^ TCRVα7.2^+^ CD161^+^) and their subsets (CD4^+^, CD8^+^ and CD4^−^CD8^−^) was evaluated in peripheral blood of individuals with VL (
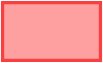
, VL, n = 8), VL–HIV co-infection (
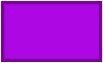
, VL–HIV, n = 10) and VL–HIV coinfected patients under liposomal amphotericin B treatment (
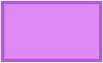
, VL–HIV_(T)_, n = 9), as well as reference groups comprising HIV-mono-infected individuals (HIV, n = 11) and non-infected individuals (NI, n = 11). The results are presented as the scattering distribution of individual values over bars underscoring the median values and interquartile range of gated lymphocytes. Comparative analyses of the VL, VL–HIV and VL–HIV_(T)_ groups as compared to NI and HIV groups were performed by the Mann-Whitney test. The dashed lines represent the confidence intervals of NI and HIV groups in green and blue, respectively. Significant differences are represented by * or ** to denote the p-values <0.05 or <0.01, respectively. **(B)** Flow cytometry gating strategy of time, singlets, lymphocytes and CD3^+^ T cells. **(C)** Flow cytometry gating strategy of organizational chart to define MAIT cells. **(D)** Flow cytometry gating strategy of MAIT subsets in each group were carried out as described in Population, Material and Methods section. Statistical significance was assessed using the Mann–Whitney U test. Y-axis is displayed on a logarithmic scale to allow visualization of populations with a wide range of frequencies.

**Figure 3:**
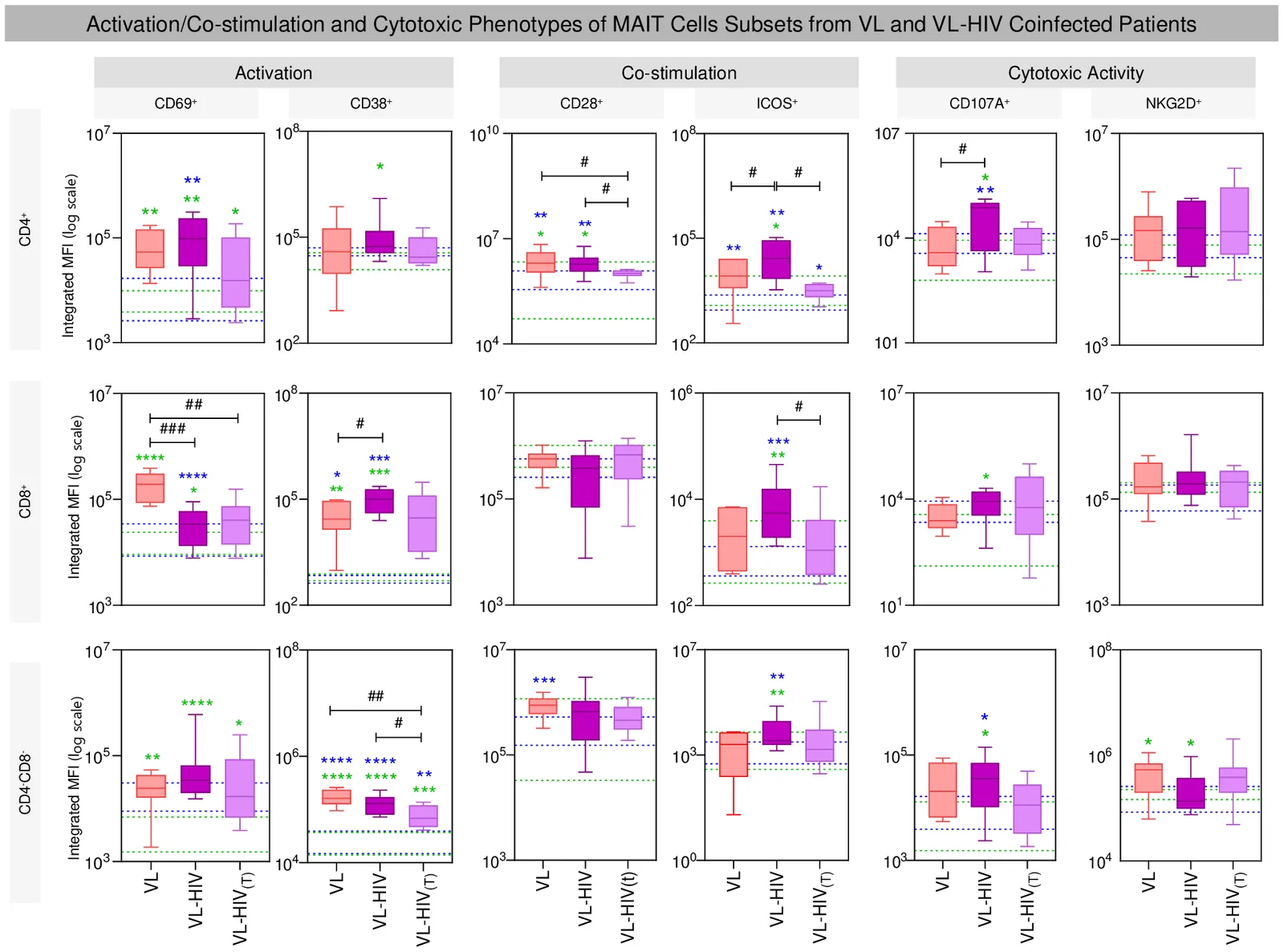
Activation/co-stimulation and cytotoxic phenotypes of MAIT cells subsets from VL and VL–HIV coinfected patients. The activation/co-stimulation and cytotoxic phenotypes profiles of CD4^+^, CD8^+^ and CD4^−^CD8^−^ MAIT cell subsets were characterized in peripheral blood of individuals with VL (
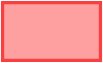
, VL, n = 8), VL–HIV co-infection (
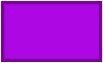
, VL–HIV, n = 10) and VL–HIV coinfected patients under liposomal amphotericin B treatment (
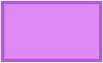
, VL–HIV_(T)_, n = 9), as well as reference groups comprising HIV-mono-infected individuals (HIV, n = 11) and non-infected individuals (NI, n = 11). The results are expressed as integrated Mean Fluorescence Intensity (iMFI) and presented in boxplot format, indicating the median values (min-max values and interquartile range 25^th^–75^th^). Comparative analyses were carried out by the Mann–Whitney test. Significant differences are represented by connecting lines with hash symbols (# p < 0.05; ## p < 0.01; ### p < 0.001), and by asterisks (*p < 0.05; **p < 0.01; ***p < 0.001; ****p < 0.0001). Green and blue asterisks indicate comparisons with the NI and HIV groups, respectively, while dashed lines represent their corresponding confidence intervals. Statistical significance was assessed using the Mann–Whitney U test. Y-axis is displayed on a logarithmic scale to allow visualization of populations with a wide range of frequencies.

**Figure 4:**
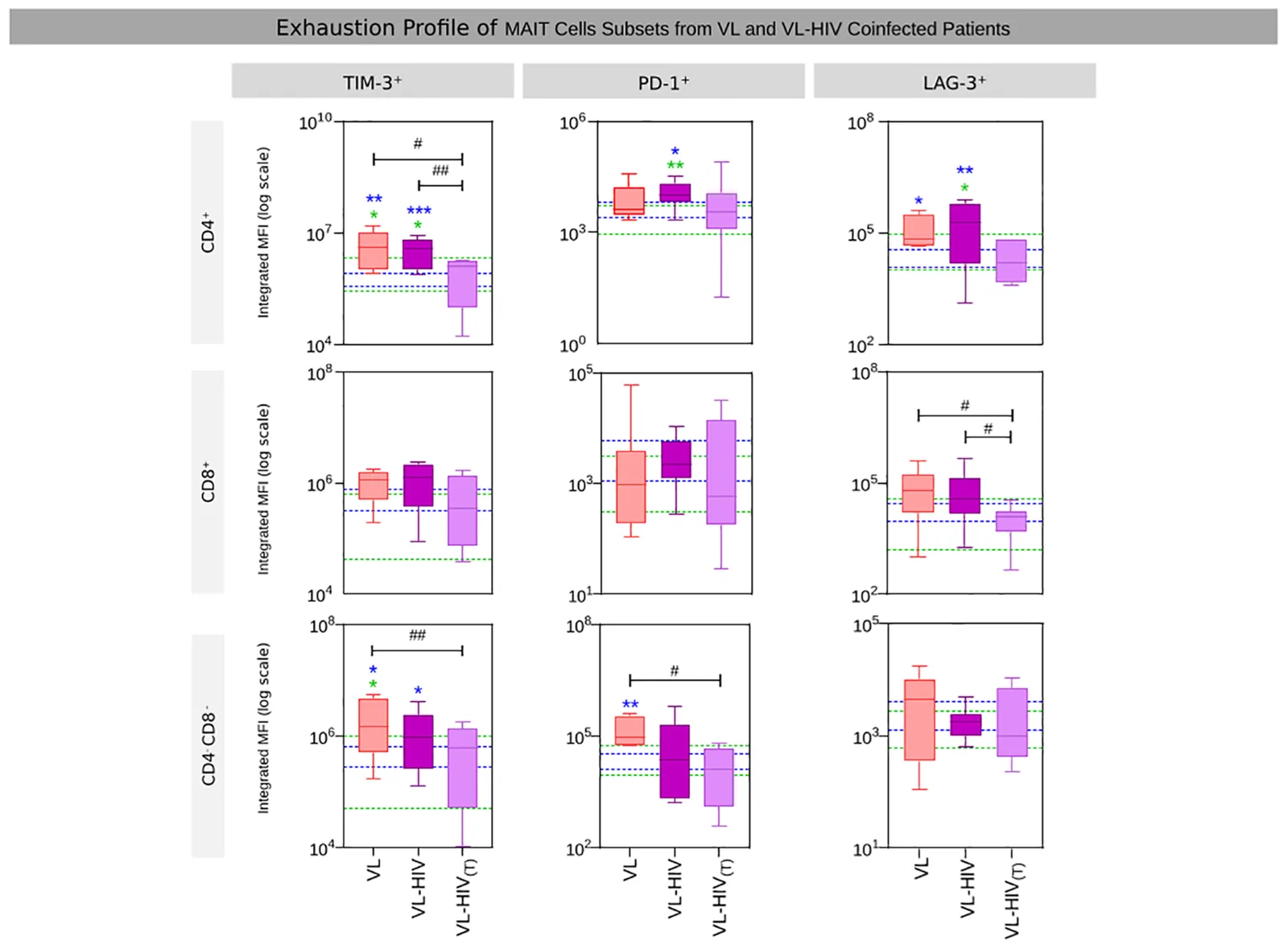
Exhaustion profile of MAIT cells subsets from VL and VL–HIV coinfected patients. The exhaustion phenotypes profiles of CD4^+^, CD8^+^, and CD4^−^CD8^−^ MAIT cell subsets (TIM-3^+^, PD-1^+^ and LAG-3^+^), was evaluated in the peripheral blood of individuals with VL (
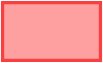
, VL, n = 8), VL–HIV co-infection (
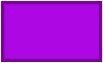
, VL–HIV, n = 10) and VL–HIV coinfected patients under liposomal amphotericin B treatment (
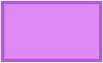
, VL–HIV_(T)_, n = 9), as well as reference groups comprising HIV-mono-infected individuals (HIV, n = 11) and non-infected individuals (NI, n = 11). The results are expressed as integrated Mean Fluorescence Intensity (iMFI) and presented in boxplot format, indicating the median values (min-max and interquartile range 25^th^–75^th^). Comparative analyses were carried out by the Mann–Whitney test. Significant differences are represented by connecting lines with hash symbols (# p < 0.05; ## p < 0.01; ### p < 0.001), and by asterisks (*p < 0.05; **p < 0.01; ***p < 0.001). Green and blue asterisks indicate comparisons with the NI and HIV groups, respectively, while dashed lines represent their corresponding confidence intervals. Statistical significance was assessed using the Mann–Whitney U test. Y-axis is displayed on a logarithmic scale to allow visualization of populations with a wide range of frequencies.

**Figure 5:**
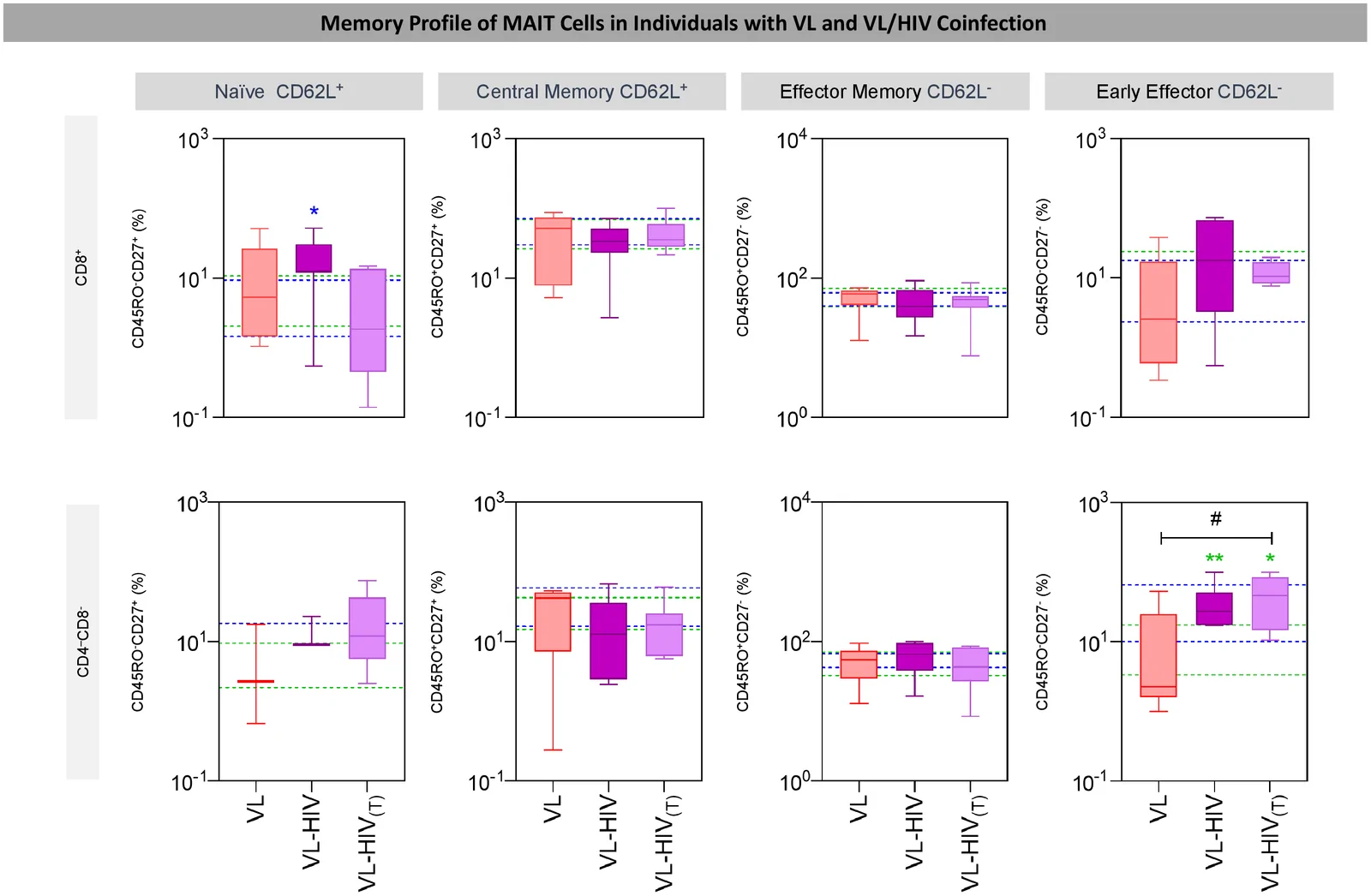
Memory profile of MAIT cells in individuals with VL and VL–HIV co-infection. The memory phenotypes profiles of naive (CD62L^+^CD45RO^−^CD27^+^), central memory (CD62L^+^CD45RO^+^CD27^+^), effector memory (CD62L^−^CD45RO^+^CD27^−^), and early effector (CD62L^−^CD45RO^−^CD27^−^) MAIT cells (CD8^+^ and CD4^−^CD8^−^) was evaluated in peripheral blood of individuals with VL (
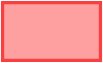
, VL, n = 8), VL–HIV co-infection (
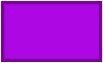
, VL–HIV, n = 10) and VL–HIV coinfected patients under liposomal amphotericin B treatment (
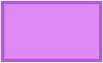
, VL–HIV(T), n = 9), as well as reference groups comprising HIV-mono-infected individuals (HIV, n = 11) and non-infected individuals (NI, n = 11). The results are expressed as integrated Mean Fluorescence Intensity (iMFI) and presented in boxplot format, indicating the median values (min-max values and interquartile range 25^th^–75^th^). Significant differences are represented by connecting lines with hash symbols (# p < 0.05; ## p < 0.01; ### p < 0.001), and by asterisks (***p < 0.001; ****p < 0.0001). Green and blue asterisks indicate comparisons with the NI and HIV groups, respectively, while dashed lines represent their corresponding confidence intervals. Statistical significance was assessed using the Mann–Whitney U test. Y-axis is displayed on a logarithmic scale to allow visualization of populations with a wide range of frequencies.

Statistical analyses were performed using a combination of software platforms to ensure robust and comprehensive data interpretation. Initially, a Time versus SSC-A plot was used to exclude signal instability and acquisition interruptions, followed by FSC-H versus FSC-A plots to identify singlets. Lymphocytes were then selected using FSC-A versus SSC-A plots, and CD3^+^ T cells were identified. MAIT cells were subsequently defined based on the co-expression of TCRVα7.2 and CD161, identifying the CD3^+^ TCRVα7.2^+^ CD161^+^. Within the MAIT gate, three subsets were distinguished according to CD4 and CD8 expression: CD4^+^, CD8^+^, and CD4^−^CD8^−^ (double-negative) MAIT cells. The phenotypic characterization included the assessment of activation/co-stimulation and cytotoxic activity (CD69, CD38, CD28, ICOS, CD107A, NKG2D), exhaustion markers (TIM-3, PD-1, LAG-3) and memory profiles (CD62L, CD45RO, CD27). Data were analyzed using FlowJo™ v10.8 (BD Biosciences). Descriptive statistical analyses were performed, and data normality was assessed by the Shapiro-Wilk test in Prism 8.0 software (GraphPad software, San Diego, USA). Comparisons between groups were carried out using the Mann-Whitney U test and with statistical significance was determined based on p-values, with p < 0.05 considered significant. Correlation analyses were performed using Spearman tests, according to data distribution. Multiple-comparison correction was performed using Bonferroni test. Fold-change (FC) values were to quantify the magnitude of changes in phenotypic, activation/co-stimulation, cytotoxicity, exhaustion, and memory marker expression in circulating MAIT cells among the study groups. FC values were calculated as the ratio between the median values of each MAIT cell marker in the reported group divided by the median values reported for the counterpart comparative group (VL vs. NI; VL vs. VL–HIV; VL vs. VL–HIV_(T)_; and VL–HIV vs. VL–HIV_(T)_). The magnitude of changes was determined considering decreases (FC < 1.0) or increases (FC > 1.0) relative to the ratio between the median values of each group and underscored by colored/gray symbols. This strategy was based in the descriptive analyses (Mann–Whitney U test) performed at **Figures 2**–**5**.

Correlation analyses were performed using Spearman correlation tests to assemble integrative networks. Only moderate and strong (“r” scores > |0.67|) significant correlations (p<0.05) were considered. Cytoscape software (available at https://cytoscape.org) was employed to build circular layouts to display three MAIT cell biomarker clusters, comprising 42 nodes representing: CD4^+^ MAIT cells (nodes 1–14), CD8^+^ MAIT cells (nodes 15–28), and CD4^−^CD8^−^ MAIT cells (nodes 29–42).

A percentile-based approach (10^th^, 50^th^, and 90^th^ percentiles) was used to represent the number of correlations within each group. The color gradient therefore reflects the distribution and intensity of network connectivity rather than individual correlation coefficients.

## 3 Results

### 3.1 Overall profile of circulating MAIT cell subsets in VL and VL–HIV coinfected patients

To characterize the overall profile of circulating MAIT cell subsets, *ex vivo* flow cytometric analysis of peripheral blood samples was performed in patients with VL, VL–HIV co-infection, VL–HIV_(T)_ as well as NI and HIV reference groups. This analysis aimed to evaluate total MAIT cell frequencies (CD3^+^ TCRVα7.2^+^ CD161^+^) and to assess their distribution among CD4^+^, CD8^+^ and CD4^−^CD8^−^ subsets, as shown in **Figure 2**. Comparative analysis demonstrated that total MAIT cell frequencies were significantly reduced in VL and VL–HIV patients compared with non-infected individuals, whereas no significant difference was observed in the VL–HIV_(T)_ group (**Figure 2A**). Analysis of MAIT cell subset distribution revealed distinct frequencies among VL groups. VL patients exhibited a relative increase of CD4^+^ MAIT cells alongside decrease of the CD8^+^ MAIT cells frequencies compared with NI individuals. In VL–HIV coinfected patients, this profile was further intensified, with reductions in CD4^+^ MAIT cells relative to HIV-mono-infected individuals, while no consistent significant difference was observed in comparison to non-infected (NI) individuals. In addition, CD8^+^ MAIT cells frequency were reduced as compared to NI. Notably, an increased frequence of CD4^−^CD8^−^ MAIT cells was consistently observed across all VL groups (VL, VL–HIV and VL–HIV _(T)_) when compared to HIV-mono-infected individuals. Representative plots depicting the gating strategy for identification of total MAIT cells and their subsets are provided in **Figure 2B**.

### 3.2 Activation/co-stimulation and cytotoxic phenotypes of MAIT cells subsets from VL and VL–HIV coinfected patients

The activation/co-stimulation and cytotoxic profiles of MAIT cells subsets were further evaluated and the results are expressed as integrated mean fluorescence intensity (iMFI), as shown in **Figure 3**. Data analysis demonstrated that activation markers (CD69 and CD38) were upregulated in VL groups, with higher iMFI values in VL and VL–HIV patients as compared to NI and HIV reference groups in the MAIT cell subsets. CD69 expression in CD8^+^ MAIT cells was increased in VL patients as compared to both VL–HIV and VL–HIV_(T)_ groups. For CD38, CD8^+^ MAIT cells from VL–HIV patients showed higher expression as compared to VL, and both VL and VL–HIV groups exhibited increased CD38 levels as compared to VL–HIV_(T)_ within the CD4^-^CD8^-^ MAIT cell subset.

Co-stimulatory marker expression revealed distinct patterns for CD28 and ICOS. CD28 expression was elevated in CD4^+^ MAIT cells from both VL and VL–HIV patients as compared to NI and HIV reference groups; both groups also showed higher CD28 levels compared with VL–HIV_(T)_ patients. Within the CD4^−^CD8^−^ subset, VL patients displayed elevated CD28 expression compared with the HIV group, with no further significant differences in the other cellular subsets. In contrast, ICOS expression was predominantly upregulated in VL–HIV patients, with significantly higher iMFI values in CD4^+^, CD8^+^, and CD4^−^CD8^−^ MAIT cells compared with both NI and HIV groups, and higher levels as compared to VL–HIV_(T)_ patients in CD4^+^ and CD8^+^ MAIT cells.

Cytotoxic activity markers also revealed distinct alterations in the MAIT cell subsets. CD107A expression was increased in MAIT cells from VL–HIV patients, with higher iMFI values observed in CD4^+^ and CD4^−^CD8^−^ MAIT cells as compared to NI and HIV reference groups. Significant difference between VL–HIV and NI groups was observed for CD8^+^ MAIT cells. VL–HIV patients displayed higher CD107A expression in CD4^+^ MAIT cells as compared to VL patients. Regarding NKG2D, higher NKG2D iMFI values were observed in CD4^−^CD8^−^ MAIT cells from VL and VL–HIV patients as compared to NI (**Figure 3**).

### 3.3 Exhaustion profile of MAIT cell subsets in VL and VL–HIV coinfected patients

To further investigate the exhaustion profile of circulating MAIT cell subsets, the expression of inhibitory immune molecules (TIM-3, PD-1 and LAG-3) was evaluated by flow cytometry and the results are expressed as integrated mean fluorescence intensity (iMFI) as shown in **Figure 4**. Overall data analysis showed increased TIM-3 expression in CD4^+^ MAIT cells from VL and VL–HIV patients as compared to NI and HIV reference groups. In CD4^−^CD8^−^ MAIT cells, TIM-3 iMFI was elevated in VL patients as compared to NI, however, no significant difference was observed between VL–HIV and NI groups in this subset. Furthermore, TIM-3 expression in CD4^+^ MAIT cells from VL and VL–HIV patients was higher as compared to VL–HIV_(T)_. In CD4^−^CD8^−^ MAIT cells, TIM-3 iMFI was elevated in VL patients as compared to VL–HIV_(T)_, while no significant difference was observed between VL–HIV and VL–HIV_(T)_. PD-1 expression was increased in CD4^+^ MAIT cells from VL–HIV patients as compared to NI and HIV groups. In CD4^−^CD8^−^ MAIT cells, PD-1 expression was elevated in VL patients as compared to HIV and VL–HIV_(T)_, while no significant difference was observed in CD8^+^ MAIT cells. LAG-3 expression was increased in CD4^+^ MAIT cells from VL and VL–HIV patients as compared to HIV group, with VL–HIV patients also showing higher levels of LAG-3 expression as compared to NI reference group. Both VL and VL–HIV patients exhibited elevated LAG-3 expression in CD8^+^ MAIT cells as compared to VL–HIV_(T)_ (**Figure 4**).

### 3.4 Memory profile of MAIT cells in VL and VL–HIV coinfected individuals

To further characterize the memory profile of circulating MAIT cell subsets, flow cytometric analysis was performed based on the expression of CD45RO, CD27 and CD62L. The memory phenotype of MAIT cells was evaluated by analyzing the frequencies of naïve (CD62L^+^CD45RO^−^CD27^+^), central memory (CD62L^+^CD45RO^+^CD27^+^), effector memory (CD62L^−^CD45RO^+^CD27^−^), and early effector (CD62L^−^CD45RO^−^CD27^−^) subsets within CD8^+^ and CD4^−^CD8^−^ MAIT cells, as shown in **Figure 5**. Due to their low frequency, CD4^+^ MAIT cells were not included in memory subset analyses to avoid unreliable sub-classification. Data analysis demonstrated increase of naïve CD8^+^ MAIT cells in VL–HIV patients as compared to HIV group. In addition, early effector CD4^−^CD8^−^ MAIT cells were increased in VL–HIV_(T)_ patients as compared to VL group. Moreover, both VL–HIV and VL–HIV_(T)_ groups exhibited higher frequencies of early effector CD4^−^CD8^−^ MAIT cells as compared to NI group. No significant difference was observed for central memory or effector memory MAIT cells in the evaluated groups (**Figure 5**).

### 3.5 Magnitude of activation, cytotoxic, exhaustion, and memory phenotypic alterations in circulating MAIT cell subsets from individuals with VL and VL–HIV co-infection

**Figure 6:**
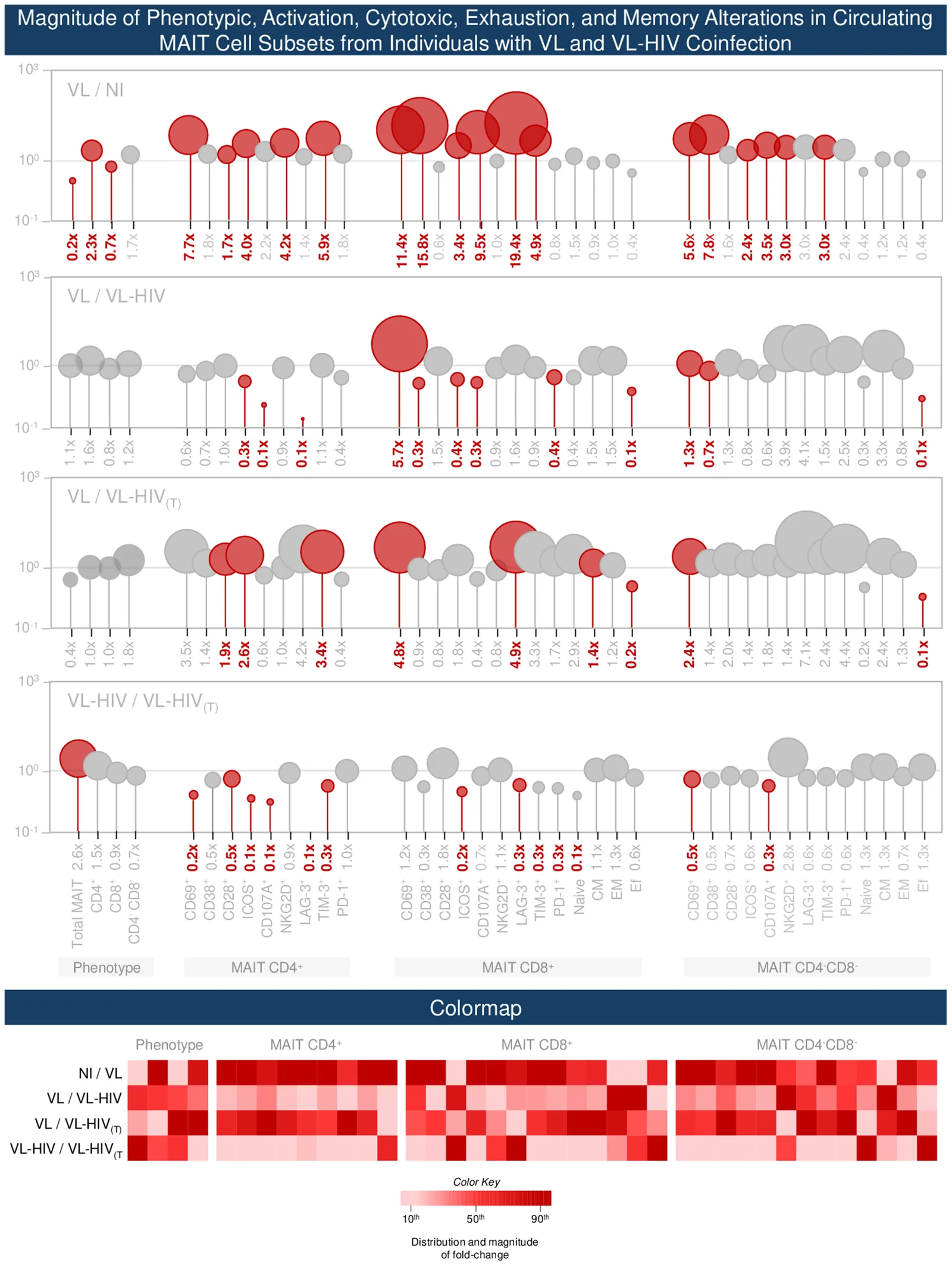
Magnitude of phenotypic, activation, cytotoxic, exhaustion, and memory alterations in circulating MAIT cell subsets from individuals with VL and VL–HIV co-infection. Fold-change (FC) analyses were performed to provide a panoramic assessment of the magnitude of alterations in circulating MAIT cell subsets across the study groups. FC values were calculated as the ratio between the median values observed in the reference group and those reported for the comparative groups, considering the following comparisons: VL/NI, VL/VL–HIV, VL/VL–HIV_(T)_ and VL–HIV/VL–HIV_(T)_. Analyses were conducted across four clusters: MAIT cell phenotype, MAIT CD4^+^, MAIT CD8^+^ and MAIT CD4^−^CD8^−^ subsets, encompassing phenotypic frequencies and the expression of activation, costimulatory, cytotoxic, exhaustion and memory markers. Increased (FC > 1.0) and decreased (FC < 1.0) values are underscored by colored symbols, as well as non-significant FC values are represented by gray symbols, according to Mann–Whitney U test. Colormap constructs summarize the distribution and magnitude of FC values across MAIT cell phenotype and subsets in all comparisons. A color key indicates the gradient of FC magnitude.

To further characterize the magnitude of phenotypic alterations in circulating MAIT cell subsets in VL and VL–HIV coinfected patients, fold change (FC) analyses were performed for VL according to NI (VL/NI), VL according to VL–HIV (VL/VL–HIV), VL according to VL–HIV_(T)_ (VL/VL–HIV_(T)_), and VL–HIV according to VL–HIV_(T)_ (VL–HIV/VL–HIV_(T)_), as shown in **Figure 6**. Overall, data analysis demonstrated that FC values ranged from 0.2x to 19.4x for VL according to NI. Reduced FC values were observed for total MAIT cells (0.2x) and MAIT CD8^+^ cells (0.7x), whereas increased FC values were detected for MAIT CD4^+^ cells (2.3x) in VL patients. Within the MAIT CD4^+^ subset, increased FC values were observed for CD69 (7.7x), CD28 (1.7x), ICOS (4.0x), NKG2D (4.2x) and TIM-3 (5.9x). In the MAIT CD8^+^ subset, increased FC values were detected for CD69 (11.4x), CD38 (15.8x), ICOS (3.4x), CD107A (9.5x), LAG-3 (19.4x) and TIM-3 (4.9x). Moreover, within the MAIT CD4^−^CD8^−^ subset, increased FC values were observed for CD69 (5.6x), CD38 (7.8x), ICOS (2.4x), CD107A (3.5x), NKG2D (3.0x) and TIM-3 (3.0x).

For VL according to VL–HIV, FC values ranged from 0.1x to 5.7x. Within the MAIT CD4^+^ subset, reduced FC values were observed for ICOS (0.3x), CD107A (0.1x) and LAG-3 (0.1x). In the MAIT CD8^+^ subset, an increased FC value was detected for CD69 (5.7x), whereas reduced FC values were observed for CD38 (0.3x), ICOS (0.4x), CD107A (0.3x), PD-1 (0.4x) and effector memory cells (EF, 0.1x). Within the MAIT CD4^−^CD8^−^ subset, an increased FC value was observed for CD69 (1.3x), along with reduced FC values for CD38 (0.7x) and effector memory cells (EF, 0.1x).

Analysis of VL–HIV_(T)_ patients compared to VL further demonstrated FC values ranging from 0.1x to 7.1x. Within the MAIT CD4^+^ subset, increased FC values were observed for CD28 (1.9x), ICOS (2.6x) and TIM-3 (3.4x). In the MAIT CD8^+^ subset, increased FC values were detected for CD69 (4.8x), LAG-3 (4.9x) and central memory cells (CM, 1.4x), whereas a reduced FC value was observed for effector memory cells (EF, 0.2x). Within the MAIT CD4^−^CD8^−^ subset, an increased FC value was observed for CD69 (2.4x), along with a reduced FC value for effector memory cells (EF, 0.1x).

The analysis of VL–HIV according to VL–HIV_(T)_ demonstrated FC values ranging from 0.1x to 2.8x. An increased FC value was observed for total MAIT cells (2.6x). Within the MAIT CD4^+^ subset, reduced FC values were detected for CD69 (0.2x), CD28 (0.5x), ICOS (0.1x), CD107A (0.1x), LAG-3 (0.1x) and TIM-3 (0.3x). In the MAIT CD8^+^ subset, reduced FC values were observed for ICOS (0.2x), LAG-3 (0.3x), TIM-3 (0.3x), PD-1 (0.3x) and naïve cells (0.1x). Moreover, within the MAIT CD4^−^CD8^−^ subset, reduced FC values were detected for CD69 (0.5x) and CD107A (0.3x). Colormap constructs were assembled to summarize the overall distribution of FC values for the groups and MAIT cell subsets, corroborating the increases and decreases identified in the FC analyses (**Figure 6**).

### 3.6 Total and selective axis networks in VL and VL–HIV patients

**Figure 7:**
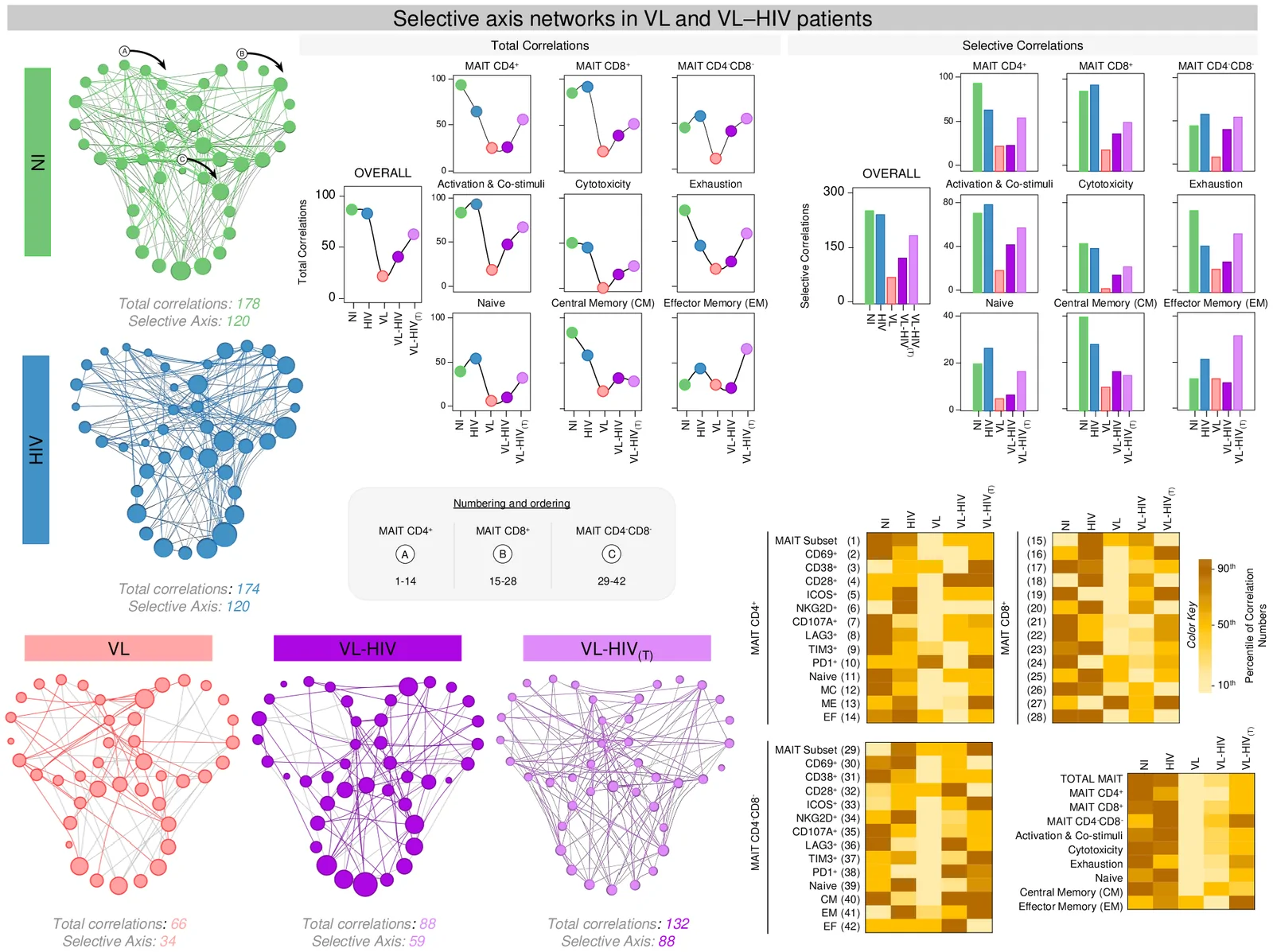
Selective axis networks in VL and VL–HIV patients. Integrative correlation networks of flow cytometry data were constructed for non-infected (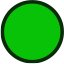, n = 11), HIV-mono-infected (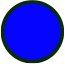, n = 11), VL (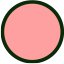, n = 8), VL–HIV (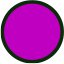, n = 10) and VL–HIV_(T)_ (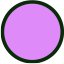, n = 9) groups using Spearman rank correlation test. Bonferroni multiple comparisons test was performed, and the adjusted p value was >0.9999 for all data. Circular network cluster layouts feature 42 nodes grouped into three clusters: MAIT CD4^+^ subset (nodes 1–14), MAIT CD8^+^ subset (nodes 15–28) and MAIT CD4^−^CD8^−^ subset (nodes 29–42). Node size is proportional to the number of significant correlations, while colored and gray edges indicate positive and negative correlations, respectively. Descriptive analyses of network connectivity, including the selective axis of correlations and the number by cluster (MAIT CD4^+^, CD8^+^ and CD4^−^CD8^−^), are shown for each group under the networks. Line graphs [central; e.g., % or MFI trends for TOTAL MAIT CD4/CD8/CD4^-^CD8^-^ across AC (activation and cytotoxic), EXHAUSTION, NAIVE, CM, EM, EF]. Bar graphs (bottom right; total, TOTAL MAIT CD4/CD8/CD4^-^CD8^-^ across AC [activation and cytotoxic], EXHAUSTION, NAIVE, CM, EM, EF across NI, HIV, VL, VL-HIV, VL-HIV_(T)_ quantify marker expression [MFI (mean fluorescence intensity)] for surface markers and phenotypes in MAIT subsets. Colormaps illustrate the distribution and density of significant correlation across MAIT cell subsets and functional clusters. The color scale represents percentile-based stratification (90^th^–10^th^ percentile) and phenotypic distributions (MFI) of the number of correlations within each group with colors ranging from light yellow to dark yellow-brown. Statistical significance was assessed using the Mann–Whitney U test.

Total and selective correlation networks in VL and VL–HIV patients were constructed using a systems biology framework to dissect the integrative interplay among parameters of MAIT cell subsets, as illustrated in **Figure 7**. Moderate-to-strong significant correlations were used to assemble integrative and selective network axes in circular layouts comprising three clusters and a total of 42 nodes (MAIT CD4^+^ (A): nodes 1–14; MAIT CD8^+^ (B): nodes 15–28; MAIT CD4^−^CD8^−^ (C): nodes 29–42).

The NI group exhibited the highest number of total correlations (n = 178), followed by HIV (n = 174), VL–HIV_(T)_ (n = 132), VL–HIV (n = 88), and VL (n = 66). Descriptive analysis of the network clusters revealed pronounced fragmentation in subset parameters, reflected by the reduced number of correlations observed in VL (n = 66) and VL–HIV (n = 88) compared with NI (n = 178). Notably, network connectivity was partially restored in the VL–HIV_(T)_ group. These trends are corroborated by the line graphs of total correlations, which show a sharp decline in connectivity in the VL and VL–HIV groups, followed by partial re-establishment in the VL–HIV_(T)_ group (**Figure 7**).

Analysis of selective connectivity axes further demonstrated that the NI and HIV groups exhibited the highest number of selective axes (n = 120 each), followed by VL–HIV_(T)_ (n = 88), VL–HIV (n = 59), and VL (n = 34), as shown in **Figure 7**. Among network clusters, descriptive analysis again revealed marked fragmentation in parameters evaluated, with substantially fewer selective correlations in VL (n = 34) and VL–HIV (n = 59) as compared to NI (n = 120). Importantly, connectivity was partially recovered in VL–HIV_(T)_ (n = 88). These patterns are reflected in the bar graphs of selective correlations, which depict a pronounced reduction in VL and VL–HIV groups and a partial restoration in the VL–HIV_(T)_ group as compared to NI and HIV groups.

These observations are further supported by the colormap, in which VL and VL–HIV patients display lighter signature colors corresponding to the 10^th^ percentile of the color scale, whereas prophylactic patients (VL–HIV_(T)_) exhibit darker tones approaching the 90^th^ percentile (**Figure 7**).

## 4 Discussion

The present study provides novel evidence that VL and VL–HIV co-infection imposes compounded and distinct dysregulation of circulating MAIT cells, characterized by quantitative depletion, phenotypic hyperactivation, functional exhaustion, and the loss of connectivity observed in interactive networks. These findings extend prior observations in mono-infection contexts and establish, for the first time, the integrated phenotypic landscape of MAIT cells considering VL and HIV co-infection. Consistent with previous reports, the present data demonstrate a marked reduction in circulating MAIT cell frequencies in both VL and VL–HIV patients relative to non-infected individuals (33, 37, 49). Such depletion is widely attributed to sustained immune activation, activation-induced cell death, tissue redistribution and disruption of immune homeostasis during chronic infection (33). Importantly, MAIT cell frequencies in VL–HIV patients under liposomal amphotericin B prophylactic treatment approximated those observed in non-infected controls, suggesting that VL is the main driver of MAIT cell depletion (30). This contrasts with the incomplete MAIT cell frequency restoration typically reported in HIV-mono-infected individuals receiving long-term antiretroviral therapy (ART) (33, 38). Together, these findings suggest that parasite-related inflammatory cues may play an important role in shaping MAIT cell homeostasis, potentially exceeding the impact of HIV infection alone, highlighting *Leishmania*-driven inflammation as a central determinant of MAIT cell depletion.

Beyond frequency cellular changes, VL patients display a relative expansion of CD4^+^ MAIT cells alongside a reduction of CD8^+^ subsets relative to NI, while VL–HIV coinfected patients show reductions in this populations primarily in comparison to HIV-mono-infected individuals. Importantly, the increase in CD4^−^CD8^−^ MAIT cells was consistently observed across all VL-coinfected individuals when compared to HIV group, suggesting that this alteration is primarily driven by visceral leishmaniasis. Similar subset differences have been described in chronic infections, where persistent antigenic stimulation drives phenotypic diversification of unconventional T-cell populations (42). Phenotypic profiling further reveals a strongly activated MAIT phenotype characterized by elevated CD69 and CD38 expression in VL patients, indicating sustained antigenic or cytokine-mediated stimulation (30, 31). Concomitant increases in CD28 expression in VL and ICOS expression in VL-HIV patients point to enhanced co-stimulatory signaling, important for T-cell differentiation (50). However, differences between CD28 and ICOS have been described, as CD28 induces IL-2 production, whereas ICOS potently induces IL-10 production, a cytokine important for suppressive function of regulatory T cells (50). Moreover, the upregulation of cytotoxic markers such as CD107A and NKG2D, particularly pronounced in VL–HIV co-infection, suggests enhanced degranulation capacity and cytolytic potential (34, 51). Together, these features support a model in which MAIT cells respond to convergent inflammatory pressures imposed by *Leishmania infantum* and HIV infection with important increase of activation/co-stimulation and cytotoxic activity (16, 30).

Nonetheless, despite this markedly activated phenotype, MAIT cells from VL and VL–HIV patients simultaneously exhibited hallmarks of functional exhaustion, as evidenced by elevated co-expression of PD-1, TIM-3, and LAG-3 (23, 52). These findings extend previous observations from our group, which demonstrated that MAIT cells in visceral leishmaniasis exhibit marked activation and functional dysregulation, along with disruption of integrative immune networks (30, 31). In this context, the increased expression of inhibitory receptors observed in the present study likely reflects a broader state of immune imbalance and functional exhaustion.

Notably, although T-cell exhaustion is predominantly described in conventional CD8^+^ T cells (53–55), the most prominent alterations in our study were observed in CD4^+^ MAIT cells. This suggests that exhaustion signatures in MAIT cells may not follow the classical paradigms established for conventional T cells, since MAIT cells are innate-like lymphocytes with substantial functional heterogeneity and distinct gene-expression programs associated with CD4^+^ or CD8^+^ co-expression (56–58). Given their innate-like nature, MAIT cells may exhibit subset-specific responses to chronic antigenic and inflammatory stimuli, particularly because prolonged stimulation can drive heterogeneous activation and exhaustion states in MAIT cells (56) and because chronic infections are associated with increased inhibitory receptor expression and dysfunction in MAIT populations (33, 59, 60). This may be especially relevant in the context of VL and VL–HIV co-infection, where persistent immune activation and chronic antigenic stimulation are linked to T-cell exhaustion, high PD-1 expression, and impaired effector responses (16, 22, 61, 62). Previous studies have shown that chronic activation of T cells, especially CD8^+^ T cells, leads to cellular exhaustion in VL (63, 64), HIV infection (65–67), and VL-HIV co-infection (62). The authors report that exhausted cells are dysfunctional and have reduced responses to pathogens (62, 68–70). Moreover, the co-expression of multiple inhibitory checkpoints is of particular concern, as polyfunctional exhaustion has been associated with advanced T-cell dysfunction and failure of pathogen control in other chronic infections (53, 67, 71, 72). Thus, this concurrent hyperactivation–exhaustion phenotype observed in this study, suggests that antigenic and cytokine-mediated stimulation drives progressive loss of effector capacity, potentially impairing sustained MAIT cell function, in line with data showing that MAIT cells in chronic HIV-1 infection are highly activated yet functionally exhausted and progressively depleted under persistent microbial stimulation (33). On the other hand, VL–HIV_(T)_ patients displayed attenuated expression of exhaustion markers, supporting the concept that parasite control is necessary and potentially sufficient to partially reverse the exhausted MAIT cell phenotype in coinfected individuals, consistent with broader evidence that reduction of chronic antigenic stimulation can facilitate partial functional recovery of exhausted T cells (73, 74). This pattern mirrors broader paradigms of immune reconstitution in chronic infection: therapeutic suppression of antigenic load leads to reduced activation and gradual restoration of T-cell function as shown for HIV, where antiretroviral therapy and interventions that dampen chronic type I interferon–driven activation decrease exhaustion marker expression and restore virus-specific CD8^+^ T-cell function (75, 76). Within this framework, MAIT cells may represent sensitive indicators of the balance between persistent immune stimulation and immune recovery, given their documented activation, functional impairment, and only partial or absent restoration despite control of the underlying chronic infection (37, 45, 77–81).

Alterations in memory-associated markers further support this interpretation. The increased frequency of naïve CD8^+^ MAIT cells in VL–HIV patients may reflect impaired differentiation or disrupted homeostatic proliferation under HIV-associated immune dysfunction, consistent with previous reports of memory maturation arrest in chronic viral infections (82–84). Conversely, enrichment of early effector CD4^−^CD8^−^ MAIT cells in VL–HIV_(T)_ patients suggest active immune restructuring following parasite control, indicative of a transitional state rather than full functional reconstitution. A derivative relationship between CD8^+^ and CD4^−^CD8^−^ MAIT cells has been demonstrated, with CD8^+^ MAIT cells giving rise to functionally distinct CD4^−^CD8^−^ MAIT cells under chronic TCR stimulation, supporting the view of CD4^−^CD8^−^ MAIT cells as an activation-induced effector subset (42).

Thus, our data indicate that VL and VL–HIV co-infection not only alter MAIT phenotypes but also profoundly disrupt immune interactions, consistent with reports of systemic immune perturbation and chronic activation in VL–HIV co-infection (16). Network analyses reveal a marked reduction in both total and selective correlations among MAIT subsets in VL and VL–HIV groups compared with non-infected or HIV-mono-infected individuals, indicative of a loss of coordinated immune circuits integrating MAIT cells with inflammatory and regulatory pathways (30). This approach captures higher-order structural properties of the immune network rather than pairwise correlation strength alone, providing a system-level perspective of immune dysregulation. This profile of immune interactions suggests breakdowns in integrated immune circuits that normally couple MAIT cells to broader cytokine and myeloid networks, in line with MR1-focused network studies showing that MR1 blockade reshapes cluster connectivity among soluble mediators in VL (31). Interestingly, VL–HIV patients under liposomal amphotericin B prophylactic treatment display only partial clinical recovery, suggesting that parasite control can enable, but does not guarantee, full immune reconstitution in this setting (85, 86). These findings align with MR1-dependent mechanistic data demonstrating that MAIT cells contribute to organization of cytokine networks and protective type-1 signatures during VL, reinforcing their role as key coordinators of integrative immune circuits (30, 31).

Study limitations should be acknowledged. First, the cross-sectional design precludes longitudinal tracking of MAIT cell dynamics during disease progression or therapeutic response; second, the modest group sizes (n = 8–11 per group), inherent to the reduced number of cases, especially VL cases observed during the study period, may limit statistical power. It is important to acknowledge that MAIT cells were defined based on CD3^+^TCRVα7.2^+^CD161^+^ expression. Although this strategy is widely used for MAIT cell identification, CD161 expression can be downregulated under conditions of chronic activation and exhaustion, such as HIV infection and visceral leishmaniasis. Therefore, this approach may underestimate MAIT cell frequencies, particularly in disease groups, and should be considered when interpreting the observed depletion of MAIT cells.

Future research integrating high-dimensional immunophenotyping and spatial transcriptomics analysis in longitudinal VL–HIV cohorts will be critical to define the relationships between MAIT cells, VL, and VL-HIV co-infection. Such approaches could clarify whether restoration of MAIT connectivity merely reflects immune improvement or actively contributes to host resistance.

## 5 Conclusion

The present study provides the first comprehensive phenotypic characterization of circulating MAIT cells in individuals with visceral leishmaniasis, stratified according to HIV co-infection status and treatment with liposomal amphotericin B. Collectively, our findings demonstrate that VL and VL–HIV co-infection impose quantitative depletion and profound qualitative dysregulation of MAIT cells, characterized by concurrent hyperactivation, exhaustion, and systems-level network fragmentation. These alterations were most pronounced in untreated coinfected individuals, reflecting the synergistic immunosuppressive burden of dual infection. Critically, liposomal amphotericin B treatment was associated with partial restoration of MAIT cell frequencies, attenuation of the hyperactivated/exhausted phenotype, and re-establishment of coordinated immunological network connectivity. Together, these findings position MAIT cells as sensitive indicators of immune disruption and potential biomarkers of treatment response in VL–HIV co-infection, with implications for monitoring immune alteration in this high-risk population.

## Data Availability

The raw data supporting the conclusions of this article will be made available by the authors, without undue reservation.
